# Entrepreneurial intention among undergraduate health sciences students: a scoping review protocol

**DOI:** 10.1016/j.mex.2026.104215

**Published:** 2026-10-18

**Authors:** Gustavo Gouveia Macedo, Emerson Roberto dos Santos, João Daniel de Souza Menezes, Matheus Querino da Silva, Ana Maria Rita Pedroso Vilela Torres de Carvalho Engel, Leonila Santos de Almeida Sasso, Bruna de Carvalho Regis, Walisson Trindade Batista, Marcia Regina Furlani, Cristiane Gouveia de Souza, Renata Dalbianco Bongardi, Laís Fernanda de Amorin, Giovanna Albefaro Sandonato Barbosa, Lucas Eduardo Luizão, Izaura dos Santos Ramos, Paula Buck de Oliveira Ruiz, Antônio Hélio Oliani, Denise Cristina Móz Vaz Oliani, Luís Cesar Fava Spessoto, Júlio César André

**Affiliations:** a Center for Studies and Development of Health Education – CEDES, São José do Rio Preto Medical School - FAMERP, Avenida Brigadeiro Faria Lima, 5416, São José do Rio Preto, São Paulo, Brazil; b Health Education Supervisor Expedicionários da Saúde (Health Expeditionaries) 674 Dona Maria Franco Salgado Ave. – Jardim Atibaia (Sousas) Campinas – SP, ZIP Code13106-290, Brazil; c University Hospital Center Cova da Beira, University of Beira Interior, 6200-251 Covilhã, Portugal

**Keywords:** Health professions education, Entrepreneurship education, Higher education, Intention, Undergraduate students

## Abstract

This article presents a scoping review protocol for mapping evidence on entrepreneurial intention (EI) among undergraduate health sciences students, with emphasis on Nursing, Medicine, and Psychology. Its contribution is methodological: it converts generic Joanna Briggs Institute (JBI) guidance into a pre-specified, reusable workflow for reviews at the interface of entrepreneurship research and health professions education, two fields with distinct vocabularies. Compared with a standard JBI protocol, it adds (i) criteria-based selection of seed articles and derivation of search terms from both literatures, using truncated stems (e.g., entrepren*) and entrepreneurship-specific terms (e.g., “new venture”); (ii) a multilingual layer with Portuguese and Spanish terms and trilingual DeCS descriptors; (iii) use of reviews only for backward and forward citation chasing; (iv) a disaggregation rule for mixed samples; (v) a reproducible grey-literature procedure; and (vi) a pre-execution step justifying the added value of the review for its population and context. Seven databases and grey literature will be searched without language or date restrictions; two independent reviewers will screen and chart data, and reporting will follow PRISMA-ScR. The protocol is a starting template for reviews on EI among health sciences students; its transferable components can be adapted to adjacent disciplines.

• Provides a reusable, pre-registered JBI/PRISMA-ScR workflow for scoping reviews at the interface of entrepreneurship research and health professions education, focused on entrepreneurial intention among undergraduate health sciences students.
• Adds a criteria-based seed-article procedure for deriving search terms from both fields, using truncated root stems (e.g., entrepren*) and entrepreneurship-specific terms (e.g., “new venture”), together with a multilingual (English, Portuguese, Spanish) search layer.
• Specifies a citation-chasing-only rule for reviews, a disaggregation rule for mixed samples, and a pre-execution step to justify the added value of the review for its population and context, indicating which components can be reused in adjacent disciplines and which must be adapted.
• Strengths: the protocol follows the JBI methodology and PRISMA-ScR reporting standards, is pre-registered on the Open Science Framework, and employs a three-step search strategy that combines controlled vocabulary descriptors and free-text keywords across seven databases, with explicit core terms per PCC component, thereby enhancing transparency and reproducibility.
• Limitations: in accordance with scoping review methodology, formal critical appraisal of the included studies will not be performed; studies with interdisciplinary or mixed samples may be excluded when data specific to undergraduate health sciences students cannot be disaggregated; heterogeneity across educational and cultural contexts may constrain the comparability of the synthesized findings; records in languages other than English, Portuguese, and Spanish that lack English metadata may be missed; and the protocol is a starting template for reviews on entrepreneurial intention among undergraduate health sciences students, so its population block and added-value justification must be redeveloped for other populations.

## Graphical abstract

**Figure:**
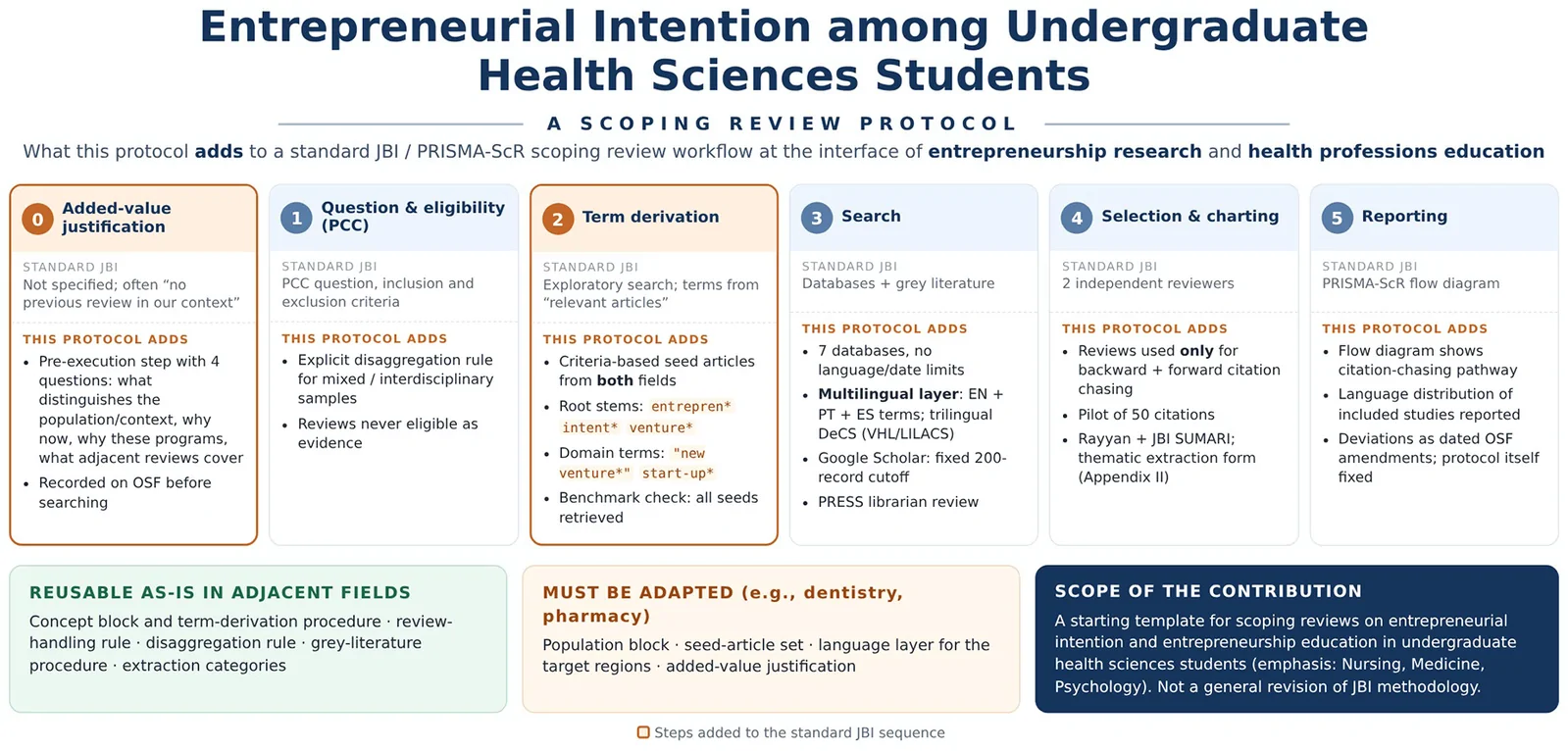

**Specifications table**

This table provides general information on your protocol.

**Table:** 

**Subject area**	Medicine and Dentistry
**More specific subject area**	Health professions education and entrepreneurship education
**Name of your protocol**	Entrepreneurial Intention among Undergraduate Health Sciences Students: a Scoping Review Protocol
**Reagents/tools**	EndNote Web (for reference management and duplicate removal); Rayyan (Rayyan Systems Inc., Cambridge, MA, USA) (for screening); JBI SUMARI (for data management and full-text assessment)
**Experimental design**	This is a scoping review protocol conducted according to the Joanna Briggs Institute (JBI) methodology. It utilizes a three-step search strategy across seven electronic databases (PubMed, Scopus, EMBASE, Cochrane Library, VHL, LILACS, and Web of Science) and grey literature, with no language or publication date restrictions. Two independent reviewers will conduct the systematic screening and data extraction, and the results will be reported using a PRISMA-ScR flow diagram, thematic matrices, and a narrative synthesis.
**Trial registration**	Registered on April 15, 2026, in the Open Science Framework (OSF) under registration number osf.io/8bszt
**Ethics**	Not applicable. (This protocol describes a scoping review of existing literature and does not directly involve primary data collection from human subjects, animal experiments, or social media platforms).
**Value of the Protocol**	•Provides a reusable, pre-registered JBI/PRISMA-ScR workflow for scoping reviews at the interface of entrepreneurship research and health professions education, focused on entrepreneurial intention among undergraduate health sciences students. •Adds a criteria-based seed-article procedure for deriving search terms from both fields, using truncated root stems (e.g., entrepren*) and entrepreneurship-specific terms (e.g., “new venture”), together with a multilingual (English, Portuguese, Spanish) search layer. •Specifies a citation-chasing-only rule for reviews, a disaggregation rule for mixed samples, and a pre-execution step to justify the added value of the review for its population and context, indicating which components can be reused in adjacent disciplines and which must be adapted.

## Background

Contemporary healthcare systems face unprecedented structural and financial challenges, underscoring an urgent imperative for innovation, adaptive leadership, and sustainable value creation [1,2] . Within this evolving landscape, health entrepreneurship has emerged as a fundamental competency, equipping future healthcare professionals to transform clinical insights into viable societal solutions [3,4]. Entrepreneurship is increasingly recognized as a driver of innovation capacity and workforce adaptability in healthcare, as health systems worldwide pursue sustainable models of value creation [5]. Entrepreneurial intention (EI), defined as a conscious cognitive predisposition to initiate venture creation, serves as the primary predictor of actual entrepreneurial behavior [6], and is theoretically grounded in models of planned behavior linking attitudes, perceived behavioral control, and social norms to the formation of entrepreneurial intent [7]. Despite its recognized importance, the integration of formal entrepreneurship training into higher health education remains embryonic, particularly within developing nations [8,9]. Moreover, existing literature exhibits substantial fragmentation, lacking a unified theoretical framework tailored to health disciplines, standardized measurement tools, or comprehensive syntheses of curriculum-level interventions [6,10]. Consequently, the evidence on EI among undergraduate health sciences students remains dispersed across heterogeneous studies, which constrains the formulation of evidence-informed educational policies and curricular reforms. Scoping reviews are recommended precisely for such contexts, as they allow the extent, range, and nature of the evidence on a broad topic to be mapped, key concepts to be clarified, and knowledge gaps to be identified [8,11,12]. This protocol delineates a rigorous, transparent methodology for mapping the body of knowledge surrounding EI among undergraduate health sciences students, with a primary focus on Nursing, Medicine, and Psychology. By outlining a structured framework based on international standards, this protocol establishes the operational foundation for synthesizing current evidence, clarifying core conceptual constructs, and identifying persistent knowledge gaps. Researchers and medical educators can utilize this protocol to replicate or adapt the scoping framework across related health disciplines, thereby promoting methodological harmonization in health professions education research.

The contribution of this article is methodological and deliberately bounded. Published JBI scoping review protocols usually apply the generic JBI stages to a single disciplinary literature. The present topic, however, lies at the interface of two fields: entrepreneurship research, which is largely indexed in business and management sources and uses its own vocabulary (e.g., new venture creation, start-up intention, self-employment), and health professions education, which is indexed in biomedical databases with controlled vocabularies such as MeSH, Emtree, and DeCS. The generic JBI guidance [8,9,13] specifies what each stage should achieve but leaves the corresponding operational decisions to the review team. This protocol makes those decisions explicit and reusable: a criteria-based procedure for selecting seed articles and deriving search terms from both literatures; truncated root stems and entrepreneurship-specific terms; a multilingual search layer; a citation-chasing-only rule for reviews; a disaggregation rule for mixed samples; a reproducible grey-literature procedure; and a pre-execution step in which the added value of the review for its population and context is justified. These components are summarized in the section “Methodological adaptations and transferable features”.

The protocol is intended primarily as a starting point for scoping reviews on entrepreneurial intention and entrepreneurship education among undergraduate health sciences students. Researchers in adjacent health disciplines (e.g., dentistry, pharmacy, physiotherapy, or nutrition) may reuse the concept block, the term-derivation procedure, and the review-handling and disaggregation rules, but will need to adapt the population block and to carry out the added-value justification for their own population and context.

## Description of protocol

### Research questions

This scoping review will be guided by the following main question: "What is known about the entrepreneurial intention of undergraduate students in health sciences?" The review is structured according to the PCC mnemonic, in which P (Population) comprises undergraduate students enrolled in health sciences programs, with emphasis on Nursing, Medicine, and Psychology; C (Concept) refers to entrepreneurial intention; and C (Context) encompasses higher education in health sciences.

To comprehensively address the main question and the objectives of this review, the following sub-questions were developed:

1. How is entrepreneurial intention conceptualized and defined in the literature related to health sciences higher education?
2. What factors (individual, educational, contextual) influence the entrepreneurial intention of undergraduate health sciences students?
3. What instruments and methods are used to measure entrepreneurial intention in this population?
4. What educational practices, interventions, or pedagogical strategies have been implemented to develop entrepreneurial intention in undergraduate health sciences students, and what are their outcomes?

Each of the four sub-questions is directly aligned with a corresponding objective of this review, thereby ensuring coherence between the research questions and the stated aims.

### Justification of the added value of the review (pre-execution step)

Before the searches are executed, and as a formal step of this protocol, the review team will document a structured justification of why a scoping review is needed for this specific population and context, rather than relying only on the absence of a previous review. The justification addresses four questions, which future users of this protocol should answer for their own population.

i. **What distinguishes the population and context?** Undergraduate health sciences students are trained for regulated professions, within dense, competency-based curricula with mandatory clinical placements, and under professional and ethical norms that frame the relationship between health care and profit differently from business, engineering, or technology programs. In many countries, a large proportion of graduates will work in, or alongside, publicly funded health systems. These features may shape how entrepreneurial intention is formed and expressed (e.g., autonomous private practice, social entrepreneurship, or intrapreneurship within health services), so findings obtained in general university-student or business-student samples cannot be assumed to transfer to this population.
ii. **Why now?** Entrepreneurship and innovation competencies are increasingly discussed as part of health professions education, whereas the evidence on entrepreneurial intention in this population remains dispersed and conceptually heterogeneous [6,10]. Mapping it at this stage can inform curricular and institutional decisions while they are being made.
iii. **Why the selected programs and level of training?** Nursing, Medicine, and Psychology were prioritized because they represent contrasting professional pathways to entrepreneurial activity: a profession with a long tradition of autonomous private practice (Psychology), a profession in which entrepreneurship and innovation are emerging as recognized professional competencies (Nursing), and a profession in which entrepreneurial activity ranges from private practice to technology-based ventures (Medicine). They are also the undergraduate programs offered by the authors’ institution, which allows the findings to be translated into curricular action. Other health sciences programs remain eligible, as specified in the inclusion criteria. Undergraduate students, rather than postgraduate trainees, were chosen because career intentions are formed before professional paths are consolidated, and the undergraduate curriculum is the stage at which educational interventions can be implemented at scale. We acknowledge that many technology-based health innovations originate in postgraduate research laboratories; this setting is outside the scope of the present review.
iv. **What do reviews in adjacent populations already cover?** Reviews and meta-analyses on entrepreneurial intention in general student and non-health populations (e.g., [5]) will be examined to identify which of their findings are, and which are not, transferable to health sciences students, so that the review is positioned in relation to that literature rather than duplicating it.

The answers to these four questions will be refined using the records retrieved in the preliminary search, recorded in the OSF registration before the full search is executed, and reported in the Introduction of the final review.

### Objectives

The overall (primary) objective of this scoping review is to map and analyze, in the international literature, without geographic restriction, the characteristics, influencing factors, and educational strategies related to entrepreneurial intention among undergraduate health sciences students, with a specific focus on Nursing, Medicine, and Psychology programs.

The specific (secondary) objectives are: 1. To examine how entrepreneurial intention is conceptualized and defined in the health sciences higher education literature; 2. To identify the individual, educational, and contextual factors that influence the entrepreneurial intention of undergraduate health sciences students; 3. To map the instruments and methods used to measure entrepreneurial intention in this population; 4. To describe the educational practices, interventions, and pedagogical strategies implemented to foster entrepreneurial intention in undergraduate health sciences students, together with their reported outcomes.

Inclusion and Exclusion criteria

**Table:** 

**PCC**	**Inclusion criteria**	**Exclusion criteria**
**P — Population**	Undergraduate students enrolled in health sciences programs (emphasis on Nursing, Medicine, and Psychology); other health programs included if EI data are extractable separately or the sample is predominantly health sciences undergraduates; any year, institution type, country, or region.	Postgraduate students, graduated professionals, technical/secondary students, or students from non-health fields; mixed samples from which health-sciences undergraduate data cannot be disaggregated.
**C — Concept**	Entrepreneurial intention and related constructs (attitude, motivation, propensity, interest, aspirations, mindset); studies on influencing factors, measurement instruments, or educational interventions addressing EI.	Studies addressing only actual entrepreneurial behavior without intention/predisposition; generic entrepreneurship education without measuring, assessing, or discussing EI.
**C — Context**	Health sciences higher education, undergraduate training in universities, colleges, or university centers; any country, region, language, or publication period.	Non-higher-education settings (technical, secondary, short courses); exclusively postgraduate or corporate/professional contexts; fields unrelated to health sciences.

### Types of sources

Only primary studies and the non-review sources listed in this section will be eligible for inclusion as sources of evidence. Within this boundary, an inclusive approach will be taken to study design. Quantitative studies will be eligible, including observational analytical and descriptive designs, such as cross-sectional, prospective and retrospective cohort, and correlational studies, as well as experimental and quasi-experimental designs, such as randomized and non-randomized controlled trials, pre-post studies, and interrupted time series that evaluate the impact of educational interventions on entrepreneurial intention. Qualitative studies that explore perceptions, experiences, meanings, and factors related to entrepreneurial intention will also be included, encompassing approaches such as phenomenology, grounded theory, ethnography, qualitative description, qualitative case studies, and action research, among others, and studies that combine both approaches (mixed methods) will likewise be considered, together with psychometric studies addressing the development, adaptation, validation, or application of instruments for measuring entrepreneurial intention.

Reviews of any type, including systematic, integrative, scoping, and umbrella reviews and meta-analyses, are not eligible for inclusion. They will be used only for backward citation chasing (screening their reference lists) and forward citation chasing (tracking the publications that cite them) to identify primary studies, in accordance with the JBI methodology for scoping reviews [8]. This procedure also prevents the double-counting of evidence, since the findings reported in such reviews derive from primary studies that are themselves eligible for inclusion and will be retrieved directly. Grey literature, including doctoral theses, master's dissertations, undergraduate final papers, conference proceedings, technical reports, and relevant governmental or institutional documents, will also be eligible, as will theoretical texts, opinion articles, editorials, and consensus documents that discuss conceptual or theoretical aspects of entrepreneurial intention in the context of health sciences higher education. No restriction will be placed on the language or the period of publication, given that research on entrepreneurship in health sciences education is an emerging and still-developing field of knowledge.

## Methods

This scoping review will be conducted in accordance with the Joanna Briggs Institute (JBI) methodology for scoping reviews [8,9,13]. The JBI methodology was chosen for its systematic approach to mapping the extent and nature of available evidence, identifying knowledge gaps, and clarifying key concepts related to entrepreneurial intention in undergraduate health sciences students. In addition, the reporting of this review will be guided by the PRISMA Extension for Scoping Reviews (PRISMA-ScR) checklist, in order to ensure transparent and complete reporting of the review process and its findings.

### Protocol registration

This protocol was registered on April 15, 2026 in the Open Science Framework (OSF) under registration number osf.io/8bszt, ensuring transparency and methodological rigor. As this is the initial version of the protocol, no amendments exist at the time of registration. Should any amendment become necessary during the execution of the review, for example, changes to the search strategy, eligibility criteria, or data extraction procedures, such amendments will be documented in the OSF registration together with their corresponding rationale.

To demonstrate that this review is not redundant with the existing literature, a preliminary search was conducted in March 2026 across the relevant databases using appropriate keywords derived from the PCC framework [8,9]. This initial search confirmed the absence of any registered or published systematic or scoping reviews addressing entrepreneurial intention among undergraduate health sciences students in any country or educational context. The relevant records identified during this preliminary search were retained and, in the final stage of the review, will be subjected to citation chasing to uncover additional primary studies. Although the review team is based in Brazil, the review is international in scope and no country or region is prioritized. LILACS, the VHL, and the Brazilian Digital Library of Theses and Dissertations (BDTD) were included only to reduce the under-representation of Latin American and Portuguese- and Spanish-language literature in the major international databases.

### Search strategy

The search strategy was designed to locate both published and unpublished studies and was developed in three steps, in accordance with the JBI methodology for scoping reviews. The search syntax presented in Appendix I is final and was pre-specified prior to the execution of the review. In a first step, a limited exploratory search was conducted in PubMed and Scopus to identify the words contained in the titles, abstracts, keywords, and controlled descriptors of relevant articles. The text words and index terms identified in this way informed the development of the full search strategy, which is presented in detail in Appendix I. The search strategy was pilot-tested in MEDLINE (PubMed) and Scopus, confirming the sensitivity and specificity of the Boolean block combinations and the retrieval of key benchmark articles in the field of health entrepreneurship education. The pilot search was conducted in March 2026.

**Selection of seed articles and derivation of search terms.** The exploratory search (March 2026) combined free-text expressions for entrepreneurship and entrepreneurial intention with terms for students and for health sciences programs in titles and abstracts. Seed articles were selected from the retrieved records using four pre-defined criteria: (a) primary or psychometric study; (b) sample composed of, or disaggregable for, undergraduate health sciences students; (c) entrepreneurial intention or a closely related construct assessed as a main outcome or variable; and (d) title, abstract, and author keywords available in English, so that their vocabulary could be compared. Two reviewers (G.G.M. and J.D.S.M.) applied these criteria independently, and the resulting seed set (including [3,4,6,10]) was supplemented with foundational references from the entrepreneurship literature on intention models and their measurement [5,7,14,15], so that domain-specific vocabulary that is uncommon in the health literature (e.g., “new venture”, “venture creation”, “start-up”, “self-employment”) would also be captured.

From each seed article, the two reviewers extracted the title words, abstract words, author keywords, and indexing terms (MeSH, Emtree, and DeCS), tabulated them by PCC component, and retained every term that described the population, concept, or context in at least one seed article; disagreements were resolved by consensus. Retained terms were reduced to their root stems and truncated (e.g., entrepren* for entrepreneur, entrepreneurs, entrepreneurship, and entrepreneurial; intent* for intention and intentions; venture*; start-up* and startup*; self-employ*), and exact phrases were used only where truncation would retrieve irrelevant terms or where a database does not support truncation within phrases. Finally, the candidate strategy was tested for its ability to retrieve all seed articles (benchmark validation); any seed article not retrieved prompted a review of the missing terms. The final concept block therefore combines truncated stems with entrepreneurship-specific terms such as “new venture*”, “venture creation”, “business creation”, start-up*, startup*, and self-employ*, as shown in Appendix I.

In the second step, a comprehensive search was carried out across seven electronic databases, PubMed (National Library of Medicine), Scopus (Elsevier), EMBASE (Elsevier), the Cochrane Library (Wiley), the Virtual Health Library (VHL), LILACS, and Web of Science (Clarivate Analytics). The strategy was structured around three conceptual blocks, population, concept, and context, combined with the Boolean operator “AND”, while synonyms and related terms within each block were combined with “OR”. The population block refers to undergraduate students in the health sciences (e.g., student*, undergraduate*, learner*, and program-specific terms for medical, nursing, psychology, and health sciences students); the concept block to entrepreneurial intention and related constructs (e.g., entrepren*, intent*, “new venture*”, “venture creation”, start-up*, startup*, and self-employ*); and the context block to the health sciences higher education setting. For each of the three blocks, the search strategy combined controlled vocabulary descriptors (e.g., MeSH terms in PubMed and Emtree terms in EMBASE, adapted to the specific indexing language of each database) with free-text keywords, in order to maximize both the sensitivity and the specificity of the searches. The strategy was adapted to the specific syntax of each database, and the complete strategies, with their operators, limiters, and retrieved results, are provided in Appendix I. No language or publication date restrictions were applied, in order to maximize coverage. All search filters and methodological decisions are pre-specified in this protocol: no restrictions on language, publication date, or study design will be imposed; the inclusion and exclusion criteria defined in the corresponding section will be applied uniformly across all databases and grey-literature sources; and any deviation from these pre-specified criteria, should it become necessary during execution, will be reported and justified transparently in the final review. To ensure the replicability of the review and in alignment with the updating policies of the consulted databases, a minimum interval of 20 days will be maintained between the date on which the searches are conducted and the end date of the search period. The database update time will be considered separately from the end date of the search period, so that the search period reflects the coverage of the databases at the time of execution, and the date of the last database update will be recorded and reported transparently in the final review.

**Language coverage.** Because no language restriction is applied, non-English records are addressed in three ways. First, the English free-text and controlled-vocabulary strategy retrieves non-English articles that are indexed with English titles, abstracts, or descriptors, as is the case for most records in MEDLINE, EMBASE, Scopus, and Web of Science; however, these databases do not automatically map English search terms to their foreign-language equivalents. Second, in the VHL and LILACS the strategy uses the trilingual DeCS descriptors (English, Portuguese, and Spanish) together with free-text terms in the three languages (e.g., empreend*, emprend*, “intenção empreendedora”, “intención emprendedora”, estudante*, estudiante*), and the same Portuguese and Spanish terms are applied in BDTD and Google Scholar. Third, citation chasing is independent of language. Records in other languages that lack English metadata may still be missed; this is acknowledged as a limitation, and the language distribution of the included studies will be reported so that potential language-related gaps can be interpreted.

In the third step, the reference lists of all included sources will be screened for additional studies, and grey literature will be searched in institutional repositories (such as BDTD and ProQuest) and on the websites of relevant organizations. The search syntax for these grey literature sources is provided in Appendix I. For the Google Scholar search, a reproducible procedure will be followed: the same search string used in the database searches, adapted to the Google Scholar syntax, will be applied with the results sorted by relevance; the first 200 records will be retrieved and screened against the inclusion criteria, with the search date and the exact query documented in the final report. Records beyond the 200th result will not be considered, given the practical limitations of Google Scholar's relevance ranking and its lack of a stable export function. Forward citation tracking will be performed in Google Scholar, which is sufficient for this purpose, and experts in the field will be contacted when necessary. Systematic, integrative, and scoping reviews and meta-analyses identified during the search are not eligible for inclusion and will be used only for citation chasing, both backward, by screening their reference lists, and forward, by tracking citations to them, to identify relevant primary studies, in accordance with the JBI methodology [8,9]. This citation-chasing procedure will likewise be applied to the relevant records retained from the preliminary non-redundancy search described above, so that additional primary studies can be uncovered in the final stage of the review. All searches will be conducted by two independent reviewers and documented in detail, and the strategy will be reviewed by a specialist librarian using the Peer Review of Electronic Search Strategies (PRESS) 2015 checklist [16] before its full execution. The retrieved records will then be imported into reference management software and subsequently into JBI SUMARI. The full search strategy is scheduled to be executed between October 2026 and January 2027, with the screening and data extraction phases to follow immediately thereafter; the full scoping review is expected to be completed by mid-2027. These dates refer to the execution of the review, not to the protocol. The protocol described in this article is final and will not be modified after publication; the planned dates are reported only for transparency. Any deviation that becomes necessary during execution (e.g., the addition of a database or the refinement of a search term) will be recorded as a dated and justified amendment in the OSF registration and reported in the final review, without altering the published protocol.

### Study/Source of evidence selection

After the searches are completed, all identified records will be collated and uploaded into EndNote Web for the removal of duplicates [17], and the remaining citations will be exported to Rayyan (Rayyan Systems Inc., Cambridge, MA, USA) [18] to manage the selection process. Screening will be carried out in two phases. As a pilot test, a sample of 50 citations will first be screened independently by two reviewers G.G.M. and J.D.S.M to test the inclusion and exclusion criteria; any disagreements will be discussed and the criteria refined before the full screening begins. Titles and abstracts will then be screened independently and in a blinded manner by the two reviewers, with each citation classified as “include”, “exclude”, or “maybe”.

In the second phase, the full texts of all potentially relevant citations will be retrieved through the CAPES Portal, institutional libraries, contact with the authors, or academic platforms; documents in any language will be considered, with professional translation when necessary. The full texts will be imported into JBI SUMARI [19,20] and assessed against the inclusion criteria by two independent reviewers, with Rayyan supporting the collaborative initial screening and JBI SUMARI ensuring compliance with the JBI methodology. Any disagreements arising at either phase will be resolved through discussion between the reviewers or, when consensus cannot be reached, by consulting a third reviewer M.Q.S. The reasons for excluding full-text sources will be recorded and categorized, for example, inadequate population, non-matching concept, context outside the scope of the review, ineligible study type, or full text unavailable, and documented in JBI SUMARI. The results of the search and the selection process will be reported in full and presented using a PRISMA-ScR flow diagram, [21]which will detail the number of records identified in each database, the records remaining after duplicates are removed, the records screened and excluded, the full texts assessed and excluded with their reasons, and the studies finally included. The flow diagram will also explicitly reflect the use of review articles for citation chasing, indicating the number of primary studies identified through this procedure, so that the complete selection pathway, including records derived from the preliminary non-redundancy search and from forward and backward citation chasing, is transparently documented.

### Data extraction

The data extracted from the included sources will be charted using a structured instrument developed specifically for this review (Appendix II), based on the objectives and the PCC framework. Charting will be performed independently by two reviewers, and any disagreements will be resolved through discussion or by consulting a third reviewer.

The charted data will be summarized descriptively and presented in tabular and narrative form, organized according to the central axes of the review: how entrepreneurial intention is conceptualized; the instruments used to measure it; the individual, educational, social and contextual, and health-specific influencing factors; the educational strategies and interventions described; and the identified gaps. In accordance with the JBI methodology for scoping reviews, no formal critical appraisal of methodological quality or meta-analysis will be performed, as the focus of this review is on mapping the extent and nature of the available literature.

### Data analysis and presentation

The extracted data will be synthesized descriptively in order to address the objectives and the research questions of the review, without a formal critical appraisal of methodological quality and with the focus kept on mapping the existing literature. The descriptive analysis will characterize the body of evidence in terms of its temporal and geographic distribution, the educational contexts in which the studies were conducted, the types of studies and their designs, the characteristics of the samples and participants, and the measurement instruments used. This will be complemented by a thematic analysis organized around the central axes of the review, the conceptualizations of entrepreneurial intention, the influencing factors (individual, educational, social, and health-specific), the measurement instruments, the educational interventions, the barriers and facilitators, and the knowledge gaps, with the data organized in thematic matrices that make it possible to identify patterns, trends, similarities, and divergences across studies.

The results will be presented in both tabular or graphical and narrative form. A PRISMA-ScR flow diagram will illustrate the complete search and selection process, and a set of tables will summarize the characteristics of the included studies, the measurement instruments and their psychometric properties, the educational interventions and their outcomes, and the influencing factors organized by dimension. Graphs and figures will display the temporal and geographic distribution of the studies, the distribution of study types and programs, and the frequency of the influencing factors, while conceptual maps will represent the interrelationships between factors, the theoretical models identified, and the connections between interventions and outcomes. A narrative synthesis will accompany the tabulated data and figures, contextualizing the findings in relation to the objectives of the review, describing the trends, patterns, and emerging themes, discussing convergences and divergences in the literature, identifying gaps, and highlighting the implications for practice and policy.

The results will be organized into the general characteristics of the studies, the conceptualization of entrepreneurial intention, the influencing factors by dimension, the measurement instruments, the educational strategies and interventions, the barriers and facilitators, and the gaps and future directions. No meta-analysis or formal critical appraisal will be undertaken, so that the focus remains on mapping the extent and nature of the literature and on identifying gaps. To ensure maximum transparency, completeness, and rigor in the reporting of the findings, this scoping review will be conducted and its results reported in accordance with the PRISMA Extension for Scoping Reviews (PRISMA-ScR) guidelines, including the use of the checklist and the flow diagram recommended by this extension.

### Methodological adaptations and transferable features

The generic JBI guidance specifies what each stage of a scoping review should achieve but leaves several operational decisions to the review team. This protocol makes the following decisions explicit and reusable, and they constitute its methodological contribution relative to a standard JBI scoping review protocol. First, the three-step search strategy maintains a set of explicit core terms for each PCC component consistently across all seven databases, providing a replicable template that can be adapted to other topics in health professions education. Second, a formal citation-chasing mechanism (backward and forward) is applied to systematic, integrative, and scoping reviews identified during the search, which are used exclusively to trace primary studies and thereby prevent the double-counting of evidence. Third, the grey-literature search follows a reproducible procedure, including a fixed cutoff for Google Scholar results and the documentation of the exact query and search date. Fourth, the protocol incorporates a preliminary non-redundancy search whose retained records are subsequently subjected to citation chasing. Fifth, the data extraction instrument (Appendix II) is organized into thematic categories, constituting a transferable tool for future scoping reviews in this field. Finally, the explicit rule for handling interdisciplinary and mixed samples, together with the use of thematic matrices and conceptual maps for synthesis, offers practical guidance that extends beyond the present topic.

In addition, the protocol includes three components that address recurrent weaknesses of interdisciplinary scoping reviews: (a) a criteria-based seed-article procedure that derives search terms from both the health-education and the entrepreneurship literatures, uses truncated root stems, and is checked by benchmark validation; (b) a multilingual search layer combining English terms with Portuguese and Spanish free-text terms and trilingual DeCS descriptors; and (c) a pre-execution step in which the added value of the review for its population and context is justified through four explicit questions, rather than by the absence of a previous review alone.

For researchers planning a scoping review in an adjacent field (e.g., entrepreneurial intention among dentistry or pharmacy students), the components that can be reused unchanged are the concept block and its term-derivation procedure, the review-handling rule, the disaggregation rule, the grey-literature procedure, and the data-extraction categories (Appendix II). The components that must be adapted are the population block, the seed-article set, the language layer (according to the regions of interest), and the added-value justification. The protocol does not claim to improve the JBI methodology in general; it is intended as a starting template for scoping reviews on entrepreneurial intention and entrepreneurship education among health sciences students, and its value outside this topic is limited to the transferable components listed above.

## Protocol validation

The rigorous methodological design of this protocol is validated through pre-registration on the Open Science Framework (OSF registration: osf.io/8bszt). Preliminary pilot testing of the search strategy, conducted in March 2026 in MEDLINE (PubMed) and Scopus verified the sensitivity and specificity of the Boolean block combinations, confirming the retrieval of key benchmark articles in the field of health entrepreneurship education. Furthermore, an initial exploratory search across MEDLINE, the Cochrane Database of Systematic Reviews, and JBI Evidence Synthesis, also conducted in March 2026, confirmed the absence of any registered or published systematic or scoping reviews addressing this specific topic within the Brazilian and broader international educational contexts. The full search strategy is scheduled to be executed between October 2026 and January 2027, with the screening and data extraction phases to follow immediately thereafter; the full scoping review is expected to be completed by mid-2027.

## Limitations

**Methodological Quality Assessment:** In accordance with standard JBI scoping review guidelines, formal critical appraisal of the methodological quality or risk of bias of included primary studies will not be performed, as the principal goal is mapping the breadth of existing evidence. Consequently, the findings will reflect the methodological quality of the underlying studies without a formal evaluation of this quality.

**Data Granularity in Interdisciplinary Samples:** Studies utilizing combined student samples (e.g., mixing undergraduate health students with business students or postgraduate trainees) may be excluded if data specific to undergraduate health sciences programs cannot be disaggregated. This may result in the omission of relevant evidence reported within broader samples.

**Institutional and Curricular Heterogeneity:** Variances in educational structures, cultural norms surrounding entrepreneurship, and health system frameworks across different geographic regions may present challenges when synthesizing and comparing pedagogical strategies universally.

**Reliance on the reporting of the included sources.** The charted data will depend on the completeness and accuracy of the information reported in the published sources. Missing or incomplete data in the original articles may not be fully retrievable, even after contact with the authors, which may introduce gaps in the mapped evidence.

**Scope of the grey literature and citation searching.** Although no language or publication date restrictions will be applied, the grey literature search is limited to specific repositories and to the first 200 records retrieved in Google Scholar, and citation chasing depends on the completeness of the reference lists of the sources examined. Some relevant evidence may, therefore, not be captured.

**Focus on selected health sciences programs.** The emphasis on Nursing, Medicine, and Psychology may not fully capture the diversity of all health sciences programs, potentially limiting the generalizability of the mapping to other health professions. Postgraduate trainees and research laboratories, where many technology-based health innovations originate, are outside the scope of the review.

**Language coverage.** Although no language restriction is applied, non-English records that lack English titles, abstracts, or descriptors may not be retrieved by the English-language strategy in the international databases. Portuguese and Spanish terms partially offset this limitation for Latin American and Iberian sources, but literature in other languages may remain under-represented.

**Scope of the protocol’s contribution.** This protocol was designed as a starting template for scoping reviews on entrepreneurial intention and entrepreneurship education among undergraduate health sciences students. Its transferable components can be reused in other fields, but the population block, the seed-article set, and the added-value justification are specific to this population and must be redeveloped for other populations or for postgraduate and professional settings.

## CRediT author statement

Conceptualization: G.G.M., J.C.A., P.B.O.R., A.M.R.P.V.T.C.E.; Data curation: M.R.F., C.G.S., R.D.B., I.S.R.; Formal analysis: E.R.S., L.C.F.S., W.T.B.; Funding acquisition: A.H.O., D.C.M.V.O.; Investigation: J.D.S.M., M.Q.S., L.S.A.S., B.C.R., G.A.S.B.; Methodology: G.G.M., E.R.S., P.B.O.R., L.E.L.; Project administration: J.C.A., A.H.O.; Resources: I.S.R., L.F.A., W.T.B.; Software: L.E.L., M.Q.S.; Supervision: A.H.O., D.C.M.V.O., L.C.F.S., J.C.A.; Validation: B.C.R., C.G.S., R.D.B.; Visualization: G.A.S.B., L.F.A., M.R.F.; Writing – original draft: G.G.M., E.R.S., J.D.S.M., A.M.R.P.V.T.C.E., L.S.A.S.; Writing – review & editing: A.H.O., D.C.M.V.O., L.C.F.S., J.C.A., P.B.O.R.

## Declaration of competing interest

The authors declare that they have no known competing financial interests or personal relationships that could have appeared to influence the work reported in this paper.

## Data Availability

No data was used for the research described in the article.
