# Supplementary material for: Entrepreneurial intention among undergraduate health sciences students: a scoping review protocol
Source: MethodsX. 2026 Oct 18;17:104215. doi: 10.1016/j.mex.2026.104215 (PMC13744212; doi:10.1016/j.mex.2026.104215)
Supplement: Supplementary file 1 [file mmc1.docx]

*Entrepreneurial Intention among Undergraduate Health Sciences Students: a Scoping Review Protocol*

**Appendix I. Search strategies**

This appendix reports the final, pre-specified search strategies for the seven electronic databases and the grey-literature sources described in the Search strategy section. The strategies combine three conceptual blocks (Population, Concept, and Context) with the Boolean operator AND, while synonyms within each block are combined with OR. Controlled vocabulary (MeSH, Emtree, and DeCS) is combined with free-text terms in the title, abstract, and keyword fields. No language, publication date, or study design limits are applied in any source. The date of execution and the number of records retrieved from each source will be reported in the PRISMA-ScR flow diagram of the final review.

**I.1 Search conventions**

| **Convention** | **Application in this strategy** |
| --- | --- |
| Truncation | Root stems are truncated (e.g., entrepren* retrieves entrepreneur, entrepreneurs, entrepreneurship, entrepreneurial; intent* retrieves intent, intention, intentions; student* retrieves student, students). The truncation symbol is * in all sources. Google Scholar does not support truncation, so explicit word forms are used there. |
| Phrases | Exact phrases are used only where truncation of a single word would retrieve irrelevant records (e.g., "new venture*", "venture creation", "business creation"). Phrase truncation is used where the interface supports it. |
| Hyphenated terms | Hyphenated and non-hyphenated forms are both included (e.g., "start-up*" and startup*; "self-employ*" and "self employ*"). |
| Health-profession entrepreneurship | Because health students often express entrepreneurial intention as the intention to open a private or own practice without using the word "entrepreneur", the concept block includes the combination (private practice OR own practice OR own business OR business ownership) AND intent*. |
| Languages | English free-text terms and controlled vocabulary are used in PubMed, EMBASE, Scopus, Web of Science, the Cochrane Library, and ProQuest. Portuguese and Spanish free-text terms are added in the VHL, LILACS, BDTD, and Google Scholar, together with the trilingual DeCS descriptors. |
| Controlled vocabulary | MeSH (PubMed, Cochrane Library), Emtree (EMBASE), and DeCS/MeSH (VHL, LILACS). Scopus searches index terms through the KEY field of TITLE-ABS-KEY; Web of Science has no thesaurus, and its Topic (TS) field covers title, abstract, author keywords, and Keywords Plus. |

**I.2 Seed articles and benchmark validation (step 1)**

Before the full search is executed, the seed set is defined using four criteria: (a) primary or psychometric study; (b) sample composed of, or disaggregable for, undergraduate health sciences students; (c) entrepreneurial intention or a closely related construct assessed as a main outcome or variable; and (d) title, abstract, and author keywords available in English. The criteria are applied independently by two reviewers. Title words, abstract words, author keywords, and indexing terms of each seed article are tabulated by PCC component, and any term not yet covered by the strategy is added. The seed set is complemented with foundational references from the entrepreneurship literature (Table I.1) to capture domain-specific vocabulary such as new venture, venture creation, start-up, and self-employment.

**Table I.1.** Primary studies cited in the protocol that meet the seed criteria, and foundational references used as sources of entrepreneurship vocabulary.

| **Ref.** | **Source** | **Role** |
| --- | --- | --- |
| [3] | Abousoliman et al., 2023 – nursing students (Egypt) | Seed article (benchmark) |
| [4] | Angeles, 2024 – undergraduate health sciences students (Philippines) | Seed article (benchmark) |
| [6] | Romero-Galisteo et al., 2022 – undergraduate health sciences students (Spain) | Seed article (benchmark) |
| [10] | Lim et al., 2021 – nursing students (Republic of Korea) | Seed article (benchmark) |
| [5] | Tsou et al., 2023 – meta-analysis of the intention–behavior relationship | Vocabulary source only (reviews are not eligible for inclusion) |
| [7] | Ajzen, 1991 – theory of planned behavior | Vocabulary source |
| [14] | Krueger et al., 2000 – competing models of entrepreneurial intentions | Vocabulary source |
| [15] | Liñán and Chen, 2009 – Entrepreneurial Intention Questionnaire | Vocabulary source |

Benchmark validation: before the full search, the strategy in each database is tested for its ability to retrieve every seed article indexed in that database. Any seed article not retrieved prompts a review of the missing terms, and the final strategy is then submitted to librarian peer review using the PRESS 2015 checklist.

**I.3 Core terms by PCC block**

| **Block** | **Controlled vocabulary (examples)** | **Free-text terms** |
| --- | --- | --- |
| Population – undergraduate students | MeSH: Students; Students, Health Occupations. Emtree: student; undergraduate student. DeCS: Estudantes; Estudantes de Ciências da Saúde / Estudiantes del Área de la Salud. | student*, undergraduate*, learner*; PT/ES: estudante*, estudiante*, graduand*, acadêmic*, académic*, discente*, universitári* |
| Concept – entrepreneurial intention and related constructs | MeSH/DeCS: Entrepreneurship (Empreendedorismo / Emprendimiento). Emtree: entrepreneurship. | entrepren*, intrapreneur*, "new venture*", "venture creation", "business creation", "start-up*", startup*, "self-employ*", "self employ*"; (private practice OR own practice OR own business* OR business ownership) AND intent*; PT/ES: empreend*, emprend*, intraempreend*, intraemprend*, "intenção empreendedora", "intención emprendedora", "novo* negócio*", "nuevo* negocio*", autoempleo |
| Context – health sciences higher education | MeSH: Education, Professional; Schools, Health Occupations; Health Occupations. Emtree: medical education; nursing education; paramedical education. DeCS: Education, Professional; Education, Medical; Education, Nursing; Health Occupations. | health*, medic*, nurs*, psycholog*, dent*, pharmac*, physiotherap*, "physical therap*", nutrition*, biomedic*, "allied health"; PT/ES: saúde, salud, enferm*, psicolog*, odontolog*, farmac*, fisioterap*, nutri* |

**I.4 Database strategies (step 2)**

**PubMed (MEDLINE)** *— National Library of Medicine, pubmed.ncbi.nlm.nih.gov*

| **#** | **Search** |
| --- | --- |
| #1 | "Students"[Mesh] OR student*[tiab] OR undergraduate*[tiab] OR learner*[tiab] |
| #2 | "Entrepreneurship"[Mesh] OR entrepren*[tiab] OR intrapreneur*[tiab] OR "new venture*"[tiab] OR "venture creation"[tiab] OR "business creation"[tiab] OR "start-up*"[tiab] OR startup*[tiab] OR "self-employ*"[tiab] OR "self employ*"[tiab] OR (("private practice"[tiab] OR "own practice"[tiab] OR "own business*"[tiab] OR "business ownership"[tiab]) AND intent*[tiab]) |
| #3 | "Education, Professional"[Mesh] OR "Schools, Health Occupations"[Mesh] OR "Health Occupations"[Mesh] OR "Psychology"[Mesh:NoExp] OR health*[tiab] OR medic*[tiab] OR nurs*[tiab] OR psycholog*[tiab] OR dent*[tiab] OR pharmac*[tiab] OR physiotherap*[tiab] OR "physical therap*"[tiab] OR nutrition*[tiab] OR biomedic*[tiab] OR "allied health"[tiab] |
| #4 | #1 AND #2 AND #3 |

**EMBASE** *— Elsevier, Embase.com*

| **#** | **Search** |
| --- | --- |
| #1 | 'student'/exp OR 'undergraduate student'/exp OR student*:ti,ab,kw OR undergraduate*:ti,ab,kw OR learner*:ti,ab,kw |
| #2 | 'entrepreneurship'/exp OR entrepren*:ti,ab,kw OR intrapreneur*:ti,ab,kw OR 'new venture*':ti,ab,kw OR 'venture creation':ti,ab,kw OR 'business creation':ti,ab,kw OR 'start-up*':ti,ab,kw OR startup*:ti,ab,kw OR 'self-employ*':ti,ab,kw OR 'self employ*':ti,ab,kw OR (('private practice' OR 'own practice' OR 'own business*' OR 'business ownership'):ti,ab,kw AND intent*:ti,ab,kw) |
| #3 | 'medical education'/exp OR 'nursing education'/exp OR 'paramedical education'/exp OR 'psychology'/de OR health*:ti,ab,kw OR medic*:ti,ab,kw OR nurs*:ti,ab,kw OR psycholog*:ti,ab,kw OR dent*:ti,ab,kw OR pharmac*:ti,ab,kw OR physiotherap*:ti,ab,kw OR 'physical therap*':ti,ab,kw OR nutrition*:ti,ab,kw OR biomedic*:ti,ab,kw OR 'allied health':ti,ab,kw |
| #4 | #1 AND #2 AND #3 |

**Scopus** *— Elsevier, scopus.com (Advanced search)*

| **#** | **Search** |
| --- | --- |
| #1 | TITLE-ABS-KEY ( student* OR undergraduate* OR learner* ) |
| #2 | TITLE-ABS-KEY ( entrepren* OR intrapreneur* OR "new venture*" OR "venture creation" OR "business creation" OR "start-up*" OR startup* OR "self-employ*" OR "self employ*" OR ( ( "private practice" OR "own practice" OR "own business*" OR "business ownership" ) AND intent* ) ) |
| #3 | TITLE-ABS-KEY ( health* OR medic* OR nurs* OR psycholog* OR dent* OR pharmac* OR physiotherap* OR "physical therap*" OR nutrition* OR biomedic* OR "allied health" ) |
| #4 | #1 AND #2 AND #3 |

**Web of Science Core Collection** *— Clarivate Analytics, all editions (Advanced search)*

| **#** | **Search** |
| --- | --- |
| #1 | TS=(student* OR undergraduate* OR learner*) |
| #2 | TS=(entrepren* OR intrapreneur* OR "new venture*" OR "venture creation" OR "business creation" OR "start-up*" OR startup* OR "self-employ*" OR "self employ*" OR (("private practice" OR "own practice" OR "own business*" OR "business ownership") AND intent*)) |
| #3 | TS=(health* OR medic* OR nurs* OR psycholog* OR dent* OR pharmac* OR physiotherap* OR "physical therap*" OR nutrition* OR biomedic* OR "allied health") |
| #4 | #1 AND #2 AND #3 |

**Cochrane Library** *— Wiley, cochranelibrary.com (all databases)*

| **#** | **Search** |
| --- | --- |
| #1 | MeSH descriptor: [Students] explode all trees |
| #2 | (student* OR undergraduate* OR learner*):ti,ab,kw |
| #3 | #1 OR #2 |
| #4 | MeSH descriptor: [Entrepreneurship] explode all trees |
| #5 | (entrepren* OR intrapreneur* OR "new venture*" OR "venture creation" OR "business creation" OR "start-up*" OR startup* OR "self-employ*" OR "self employ*"):ti,ab,kw |
| #6 | (("private practice" OR "own practice" OR "own business*" OR "business ownership") AND intent*):ti,ab,kw |
| #7 | #4 OR #5 OR #6 |
| #8 | MeSH descriptor: [Education, Professional] explode all trees |
| #9 | MeSH descriptor: [Health Occupations] explode all trees |
| #10 | (health* OR medic* OR nurs* OR psycholog* OR dent* OR pharmac* OR physiotherap* OR "physical therap*" OR nutrition* OR biomedic* OR "allied health"):ti,ab,kw |
| #11 | #8 OR #9 OR #10 |
| #12 | #3 AND #7 AND #11 |

**Virtual Health Library (VHL)** *— BIREME/PAHO/WHO, bvsalud.org (Advanced search, all sources)*

| **#** | **Search** |
| --- | --- |
| #1 | (mh:("Students" OR "Students, Health Occupations") OR tw:(student* OR estudante* OR estudiante* OR undergraduate* OR graduand* OR acadêmic* OR académic* OR discente* OR universitári* OR universitari*)) |
| #2 | (mh:("Entrepreneurship") OR tw:(entrepren* OR intrapreneur* OR empreend* OR emprend* OR intraempreend* OR intraemprend* OR "new venture*" OR "novo* negócio*" OR "nuevo* negocio*" OR "start-up*" OR startup* OR "self-employ*" OR autoempleo OR "trabalho autônomo" OR "intenção empreendedora" OR "intención emprendedora")) |
| #3 | (mh:("Education, Professional" OR "Education, Medical" OR "Education, Nursing" OR "Health Occupations") OR tw:(health* OR saúde OR salud OR medic* OR nurs* OR enferm* OR psycholog* OR psicolog* OR dent* OR odontolog* OR pharmac* OR farmac* OR physiotherap* OR fisioterap* OR nutri* OR biomedic*)) |
| #4 | #1 AND #2 AND #3 |

**LILACS** *— BIREME/PAHO/WHO, searched through the VHL portal*

| **#** | **Search** |
| --- | --- |
| #1 | (mh:("Students" OR "Students, Health Occupations") OR tw:(student* OR estudante* OR estudiante* OR undergraduate* OR graduand* OR acadêmic* OR académic* OR discente* OR universitári* OR universitari*)) |
| #2 | (mh:("Entrepreneurship") OR tw:(entrepren* OR intrapreneur* OR empreend* OR emprend* OR intraempreend* OR intraemprend* OR "new venture*" OR "novo* negócio*" OR "nuevo* negocio*" OR "start-up*" OR startup* OR "self-employ*" OR autoempleo OR "trabalho autônomo" OR "intenção empreendedora" OR "intención emprendedora")) |
| #3 | (mh:("Education, Professional" OR "Education, Medical" OR "Education, Nursing" OR "Health Occupations") OR tw:(health* OR saúde OR salud OR medic* OR nurs* OR enferm* OR psycholog* OR psicolog* OR dent* OR odontolog* OR pharmac* OR farmac* OR physiotherap* OR fisioterap* OR nutri* OR biomedic*)) |
| #4 | #1 AND #2 AND #3 AND db:("LILACS") |

In the VHL and LILACS, DeCS descriptors are entered in English through the mh: field, which retrieves records indexed with the equivalent Portuguese and Spanish descriptors; the tw: field covers title, abstract, and subject terms in all languages. Records retrieved in LILACS that are also retrieved in the VHL are removed as duplicates in EndNote Web.

**I.5 Grey literature and citation chasing (step 3)**

| **Source** | **Search procedure and syntax** |
| --- | --- |
| BDTD – Brazilian Digital Library of Theses and Dissertations (bdtd.ibict.br) | Advanced search, all fields. Portuguese string: (empreendedor* OR empreendedorismo OR intraempreend* OR "intenção empreendedora") AND (estudante* OR graduand* OR acadêmic* OR discente* OR universitári*) AND (saúde OR medicina OR enfermagem OR psicologia OR odontologia OR farmácia OR fisioterapia OR nutrição). English string: (entrepren* OR "new venture*" OR startup*) AND (student* OR undergraduate*) AND (health* OR medic* OR nurs* OR psycholog*). |
| ProQuest Dissertations & Theses Global | noft(entrepren* OR intrapreneur* OR "new venture*" OR "venture creation" OR "start-up*" OR startup* OR "self-employ*") AND noft(student* OR undergraduate* OR learner*) AND noft(health* OR medic* OR nurs* OR psycholog* OR dent* OR pharmac* OR physiotherap* OR "physical therap*" OR nutrition* OR "allied health") |
| Google Scholar (scholar.google.com) | Three strings, sorted by relevance; the first 200 records of each string are screened. Google Scholar does not support truncation and limits queries to 256 characters, so explicit word forms are used.  English: ("entrepreneurial intention" OR "entrepreneurial intentions" OR entrepreneurship OR "new venture") (students OR undergraduate) (medical OR medicine OR nursing OR psychology OR "health sciences")  Portuguese: ("intenção empreendedora" OR empreendedorismo OR empreendedor) (estudantes OR graduandos OR acadêmicos) (medicina OR enfermagem OR psicologia OR "ciências da saúde")  Spanish: ("intención emprendedora" OR emprendimiento OR emprendedor) (estudiantes OR universitarios) (medicina OR enfermería OR psicología OR "ciencias de la salud") |
| Websites of relevant organizations | Site searches and browsing of publications and reports of the Global Entrepreneurship Monitor (gemconsortium.org), the Organisation for Economic Co-operation and Development (oecd.org), the European Commission EntreComp framework (joint-research-centre.ec.europa.eu), the World Health Organization Institutional Repository (iris.who.int), and the Brazilian Micro and Small Business Support Service – Sebrae (sebrae.com.br), using the terms entrepreneurship, entrepreneurial intention, and health or medical or nursing or psychology students, and their Portuguese and Spanish equivalents. |
| Backward citation chasing | Screening of the reference lists of all included sources, of the systematic, integrative, scoping, and umbrella reviews and meta-analyses identified by the search, and of the records retained from the preliminary non-redundancy search. |
| Forward citation chasing | Google Scholar "Cited by" function applied to the same set of sources. |
| Contact with experts | Corresponding authors of included studies are contacted when full texts or disaggregated data for undergraduate health sciences students are needed. |

Search documentation: for each source, the interface, the exact query, the date of execution, and the number of records retrieved are recorded in a search log at the time of execution and reported in the final review.

**Appendix II. Data extraction (charting) instrument**

The instrument below is applied in JBI SUMARI independently by two reviewers for each included source. It is organized into thematic categories that correspond to the review objectives and the PCC framework. Fields that are not reported in a source are recorded as "not reported". In accordance with the JBI methodology, the instrument is piloted on the first sources charted and may be refined iteratively; any refinement is described in the final review.

| **Item** | **Field** | **Description / instruction** | **Response format** |
| --- | --- | --- | --- |
| **A. Source identification** | | | |
| A1 | Record ID | Identifier assigned in Rayyan/JBI SUMARI | Text |
| A2 | Authors and year | First author et al., year of publication | Text |
| A3 | Title | Full title in the original language | Text |
| A4 | Source type | Journal article; thesis/dissertation; undergraduate final paper; conference proceedings; report or governmental/institutional document; theoretical or opinion text; editorial; consensus document | Single choice |
| A5 | Language of publication | Original language of the full text | Single choice: English / Portuguese / Spanish / other (specify) |
| A6 | Route of identification | Database search; grey-literature search; backward citation chasing; forward citation chasing; preliminary non-redundancy search; expert contact | Single choice |
| A7 | Country and region | Country where the study was conducted; WHO region | Text |
| A8 | Funding and conflicts of interest | As declared by the authors | Text |
| **B. Study characteristics** | | | |
| B1 | Aim of the study | As stated by the authors | Text |
| B2 | Study design | Cross-sectional; cohort; correlational; RCT; non-randomized trial; pre-post; interrupted time series; qualitative (specify approach); mixed methods; psychometric; theoretical/opinion | Single choice |
| B3 | Data collection methods | Questionnaire, interview, focus group, document analysis, other | Multiple choice |
| B4 | Data collection period | Months/years of data collection | Text |
| B5 | Theoretical framework | Theory or model underpinning the study (e.g., theory of planned behavior, entrepreneurial event model, social cognitive career theory) | Text |
| **C. Population** | | | |
| C1 | Undergraduate program(s) | Nursing; Medicine; Psychology; other health program (specify) | Multiple choice |
| C2 | Sample size | Total participants and number per program | Number |
| C3 | Year/stage of the program | Year(s) or semester(s) of the participants | Text |
| C4 | Participant characteristics | Age (mean/SD or range), sex/gender distribution, prior entrepreneurial experience, family business background | Text |
| C5 | Mixed sample and disaggregation | Whether the sample includes non-health or postgraduate participants and whether data for undergraduate health sciences students are reported separately | Yes / no + description |
| **D. Context** | | | |
| D1 | Type of institution | Public; private; community/philanthropic; not reported | Single choice |
| D2 | Educational setting | University, college, or university center; curriculum model (e.g., traditional, problem-based, competency-based) | Text |
| D3 | Entrepreneurship content in the curriculum | Mandatory course; elective course; extracurricular activity; none; not reported | Single choice |
| D4 | Health system and labor-market context | Features of the health system or labor market described by the authors as relevant to entrepreneurship | Text |
| **E. Conceptualization of entrepreneurial intention (objective 1)** | | | |
| E1 | Definition of EI | Verbatim definition and cited source | Text (verbatim) |
| E2 | Construct(s) examined | Entrepreneurial intention; attitude; motivation; propensity; interest; aspirations; mindset; intrapreneurship; social entrepreneurship; other | Multiple choice |
| E3 | Type of entrepreneurship addressed | Business venture; private or own practice; social entrepreneurship; intrapreneurship within health services; technology-based venture; not specified | Multiple choice |
| **F. Measurement of entrepreneurial intention (objective 3)** | | | |
| F1 | Instrument name and source | Name of the instrument and original reference (e.g., Entrepreneurial Intention Questionnaire) | Text |
| F2 | Structure | Number of items, dimensions/subscales, response scale | Text |
| F3 | Adaptation and language | Original, translated, or cross-culturally adapted; language of application | Text |
| F4 | Psychometric evidence reported | Reliability (e.g., Cronbach's alpha, omega), validity evidence (content, structure, convergent), invariance | Text |
| F5 | Qualitative methods for EI | Interview guides, focus groups, or other methods used to explore EI | Text |
| F6 | EI level reported | Descriptive results for EI (e.g., mean score, proportion with high intention) | Text |
| **G. Influencing factors (objective 2)** | | | |
| G1 | Individual factors | e.g., self-efficacy, personality traits, risk-taking, need for achievement, sex/gender, age, prior experience | Text + direction of association |
| G2 | Educational factors | e.g., entrepreneurship education, curriculum, teaching methods, role models, mentoring | Text + direction of association |
| G3 | Social and contextual factors | e.g., family business background, subjective norms, economic conditions, institutional support, culture | Text + direction of association |
| G4 | Health-specific factors | e.g., professional regulation, perceived conflict between care and profit, health system structure, clinical workload, career expectations in the profession | Text + direction of association |
| G5 | Statistical approach | Analysis used to examine the factors (e.g., correlation, regression, SEM) and main effect estimates as reported | Text |
| **H. Educational practices and interventions (objective 4)** | | | |
| H1 | Intervention description | Name, content, and objectives of the practice or intervention | Text |
| H2 | Format and delivery | Course, module, workshop, incubator, project-based learning, competition, simulation, mentoring; face-to-face/online/hybrid | Multiple choice + text |
| H3 | Dose and duration | Total hours, number of sessions, duration in weeks | Text |
| H4 | Facilitators | Profile of educators (e.g., faculty, entrepreneurs, interprofessional team) | Text |
| H5 | Comparator | Control or comparison group, if any | Text |
| H6 | Outcomes and timing | Outcomes assessed (EI and related constructs) and time points | Text |
| H7 | Reported results | Main results as reported by the authors | Text |
| **I. Barriers, facilitators, and gaps** | | | |
| I1 | Barriers | Barriers to EI or to entrepreneurship education reported | Text |
| I2 | Facilitators | Facilitators reported | Text |
| I3 | Limitations reported by the authors | As stated in the source | Text |
| I4 | Gaps and recommendations | Research, educational, or policy gaps and recommendations stated by the authors | Text |
| **J. Reviewer notes** | | | |
| J1 | Key findings relevant to the review questions | Summary of the findings that answer sub-questions 1–4 | Text |
| J2 | Notes and discrepancies | Comments, discrepancies between reviewers, and how they were resolved | Text |
| J3 | Reviewer | Initials of the reviewer and date of charting | Text |

Sections E to H correspond to sub-questions 1 to 4, respectively, and their content feeds the thematic matrices and conceptual maps described in the Data analysis and presentation section.
